# Self-perceived empathic abilities of people with autism towards living beings mostly differs for humans

**DOI:** 10.1038/s41598-022-10353-2

**Published:** 2022-04-15

**Authors:** Aurélien Miralles, Marine Grandgeorge, Michel Raymond

**Affiliations:** 1 Institut de Systématique, Evolution, Biodiversité, (UMR 7205 Muséum National d’Histoire Naturelle, CNRS UPMC EPHE, Sorbonne Universités), CP30, 25 Rue Cuvier, 75005 Paris, France; 2 EthoS—Ethologie humaine et animale (UMR 6552 Univ Rennes 1), 263 av. du Général Leclerc, Bâtiment 25, 35042 Rennes cedex, France; 3 Institut des Sciences de L’Evolution de Montpellier, Univ Montpellier, CNRS, EPHE, IRD, Montpellier, France

**Keywords:** Human behaviour, Biological anthropology, Autism spectrum disorders, Phylogenetics, Evolution, Zoology

## Abstract

Being phylogenetically close involves greater empathic perceptions towards other species. To explore this phenomenon, this study investigates the influence of neurocognitive predispositions to empathy on our perceptions of other organisms. Autistic spectrum disorders (ASD) are characterized, among others, by weakened empathic skills. Our online survey involved a group of 202 raters with ASD and a control group of 1100 raters, who had to make choices to assess their empathic perceptions toward an extended photographic sampling of organisms. Results highlight that both groups present overall similar trends in their empathic preferences, with empathy scores significantly decreasing with the phylogenetic distance relatively to humans. However, the empathy score attributed to *Homo sapiens* in the ASD group represents a striking outlier in the yet very sharp overall correlation between empathy scores and divergence time, scoring our species as low as cold-blooded vertebrates. These results are consistent with previous studies, which emphasized that (1) understanding human beings would be more difficult for people with ASD than decoding “animals” and (2) that Theory of Mind impairment would not represent a global deficit in people with ASD but may relate to the mindreading of specifically human agents.

## Introduction

Despite various definitions and several different mental states related to this notion, empathy generally refers to our capability to connect with one another at an emotional level^1,2^. Our empathic faculties enable us to recognize, understand, and share the thoughts, feelings, and emotions of another. Considered as prosociality drivers, they facilitate coordination and cooperation with our fellows humans^3–6^. Our empathy can also be at work outside a strictly human relational frame since we are able to form bonds and emotionally interact with other species. In a recent study, we highlighted the evolutionary component in our empathic preferences toward other living species (i.e., ease to understand their emotions), by putting into evidence their very significant correlation with the phylogenetic proximity with us^7^. As no cultural factor (e.g., diet, ethics, beliefs, knowledge) was able to explain this phenomenon, the Anthropomorphic stimuli hypothesis was formalized to account for the sharp correlation observed: in all likelihood, the closer another organism is phylogenetically to us, the more it shares with us inherited morphological and behavioral traits (synapomorphies), and the easier it is to perceive it as an alter ego and—really or supposedly—to understand its emotions.

Like the other perceptive and neurocognitive functions, our empathic abilities result from biological evolution and are partly determined by our genes^8,9^. Our predisposition to interact emotionally with others is therefore not exclusively conditioned by experiential or cultural factors. Among the various neurodevelopmental conditions identified in humans (i.e., neurodiversity), Autistic spectrum disorders (ASD) have a significant genetic basis^10^. ASD are characterized, among others, by attention deficits in communication and social interactions, diminished interest in other people, persistent deficit in socio-emotional reciprocity and atypical sensoriality^11^. Numerous studies highlight weakened empathic skills in people with ASD, in different cultures (e.g., UK and Japan) and in both sexes^12–15^, see also the terminological clarifications provided by Fletcher-Watson & Bird in 2020^16^). The different self-report instruments used to assess empathic performances in ASD are almost exclusively involving interhuman situations^17–19^, and to our knowledge, none has ever been designed to distinguish empathy toward humans from those toward other organisms. However, decoding animals’ mental states seems to be easier for people with ASD than understanding human beings^20–25^. Several studies have shown that people with ASD can be particularly attracted by different animal species (i.e., mostly mammals^26–29^), feel attachment to them^30^, even if sometimes rather unusual fears towards animals may exist (e.g., toward cats, squirrels, frogs)^31^. Their contrasting ability to form bonds with animals has even been proposed (and then exploited) for therapeutic purposes^27,30,32–34^.

The present work proposes to investigate the influence of our biological predispositions to empathy on our perceptions of other organisms. In the continuity of a previous and related study open to everyone^7^, the present work aims to replicate the same perception test on a sample this time restricted to participants with ASD. The procedure used was identical^7^ and consisted in an online application presenting random pairs of photographs as representative as possible of the phylogenetic diversity of life (52 species of animals, plants and fungi). For each pair, the rater had to make preference choices by clicking on the photograph corresponding to the answer to the following question: “*I feel like I'm better able to understand the feelings or the emotions of* [choice among a pair of pictures representing different organisms]”. The present survey involved 202 adult participants with ASD, whose results have been compared with those obtained in the previous study open to everyone (1134 participants)^7^.

## Results

In both the ASD focal group and comparison group, all participants were 18 years or older. In the ASD focal group, people with *Asperger's Syndrome* are over-represented (67.8%). Therefore, the results presented below are mainly representing adult with high-functioning ASD, and thus do not necessarily reflect the entire population of people on the autism spectrum (see details in “Methods”).

### Similarities between raters with and without ASD

**Figure 1:**
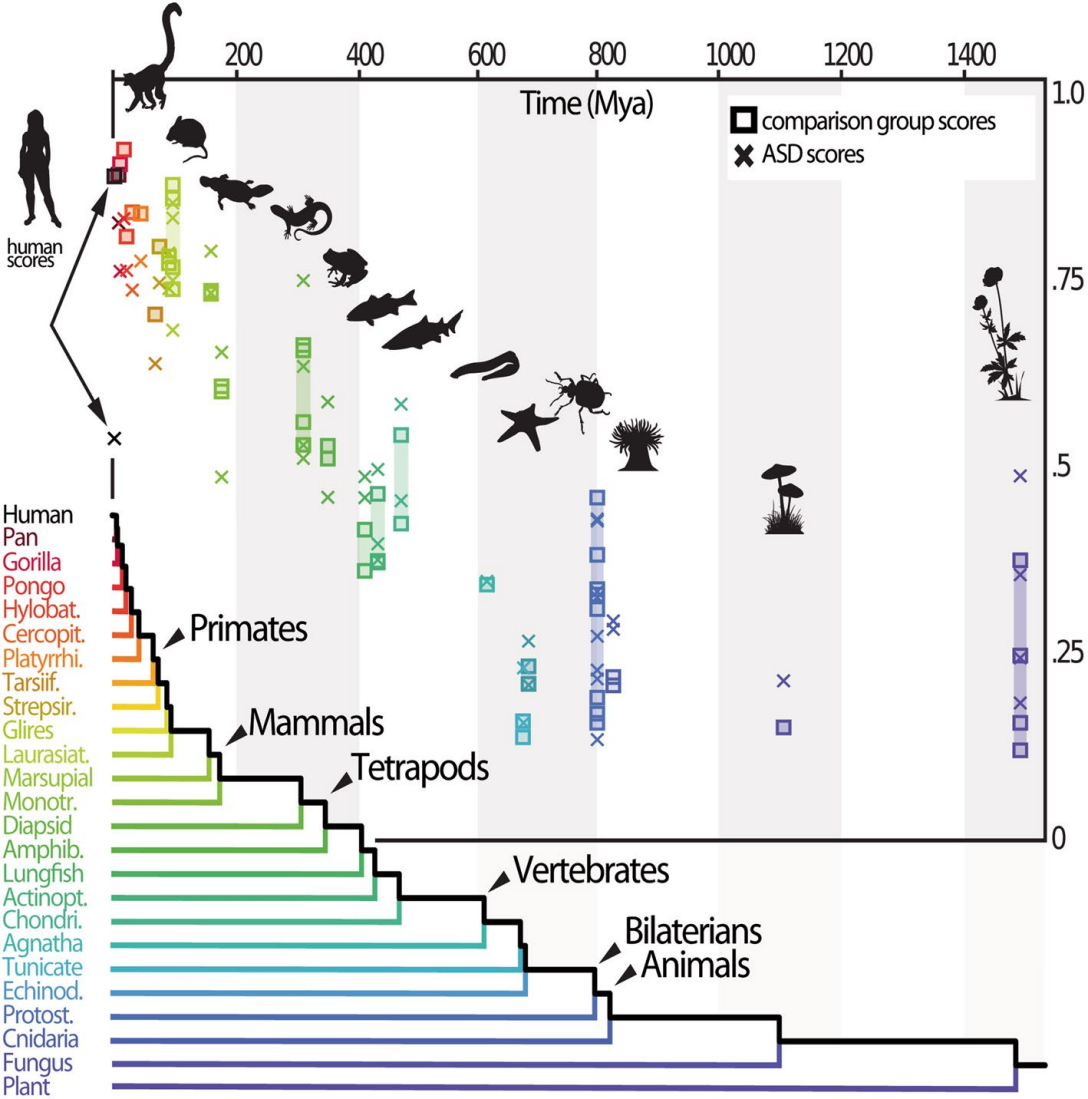
Empathy scores attributed to each organism as a function of divergence time (Mya) between them and humans. The scores correspond to the probability that a given species is chosen from a pair of species that includes it and another randomly selected (n = 52 species). Empathy scores attributed by raters in the ASD focal group and the comparison group are represented by crosses ( ×) and squares (□), respectively (cf. Supplementary S1 for more details).

Both rater’s groups present similar overall trends (Fig. 1). Like in the comparison group, the probability of a species to be chosen (i.e. based on the question “*I feel like I'm better able to understand the feelings or the emotions of […]”*) in the ASD focal group decreased with the phylogenetic distance relatively to humans, compared to the alternative species. For a relative reduction of phylogenetic distance of one million year, the probability to be chosen increased by 1.59 (SE = 0.17) in linear units (logit) for the ASD group (comparison group: 2.54, SE = 0.19). In both groups, it decreased quadratically with divergence time (ASD group: linear slope: − 1.0 10^−3^, F_1,49_ = 119, P < 10^−13^; quadratic term: 4.5 10^−7^, F_1,49_ = 49.2, P < 10^−8^; Comparison group: linear slope: − 1.2 10^−3^, F_1,49_ = 258, P < 10^−16^; quadratic term: 5.3 10^−7^, F_1,49_ = 99.8, P < 10^−13^). The empathy scores per clade obtained in both ASD and comparison groups were linearly correlated (Pearson's product-moment correlation = 0.99, t = 30.3, df = 22, P < 10^–10^, human clade excluded), with a slope (0.81, SE = 0.027) significantly different from 1 (F_1,22_ = 50.4, P = 4. 10^–7^, human clade excluded). The empathy score computed for each species, varied from 0.84 (highest score, Fox) to 0.14 (lowest score, Tick) in the ASD focal group, and from 0.91 (Orangutan) to 0.12 (Rockweed) in the comparison group (Fig. 1, Supplementary Methods S1). In both groups, the human score was significantly lower than the maximum score (ASD group: *P* < 10^–8^; comparison group: *P* < 0.01). In both ASD and comparison group, the mean response time of raters decreases significantly with the absolute time of divergence between two organisms (Supplementary Figure S4). It decreases by 0.130 s (SE = 0.022) for each increase of divergence time of 100 Myr in the ASD group (comparison group: 0.182 s, SE = 0.016). When the two species in the pair tended to be equally divergent (absolute time of divergence = 0), mean response time tended to 6.42 s (SE = 0.216) in the ASD group (comparison group: 6.61 s, SE = 0.16). Considering rater’s individual traits, results in the ASD group varied only according to the raters’ opinion on the value of animal life relatively to humans (P = 0.005), whereas in the comparison group (a dataset more than five times larger than the ASD group), they varied not only accordingly to this judgment value (P < 0.001), but also accordingly to rater’s sex (P = 0.02) and age (P < 0.001). Direction of effects for these rater’s individual traits are depicted in Supplementary Tables S5 and S6).

### Differences between raters with and without ASD (Figs. 1, 2, 3)

**Figure 2:**
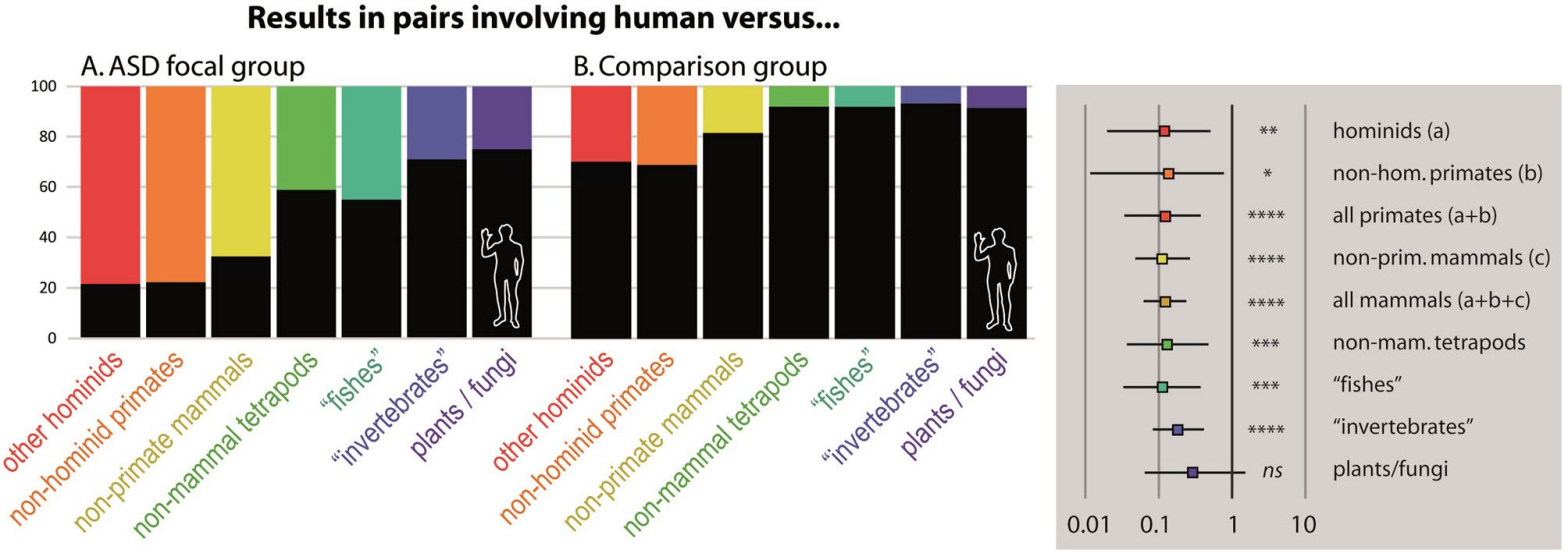
Results of photographic confrontations between a human and various other taxa (Choices in percentages, 171 pairs of photographs in total in the ASD group and 872 in the comparison group). Differences between ASD and comparison groups preferences are statistically supported for each of these choices categories, except for the pairs involving non-animal taxa, i.e., plant and fungi (Odd ratios with 95% confidence interval and significance of the Exact Fischer’s exact test are represented in the grey box : ns (non-significant) = P > 0.05, * = P ≤ 0.05, ** = P ≤ 0.01, *** = P ≤ 0.001, **** = P ≤ 0.0001). Contrary to the comparison group, a majority of raters with ASD considered themselves as *better able to understand the feelings or the emotions* in photographs depicting non-human mammals than in photographs of human beings (cf. details in Appendix S7).

**Figure 3:**
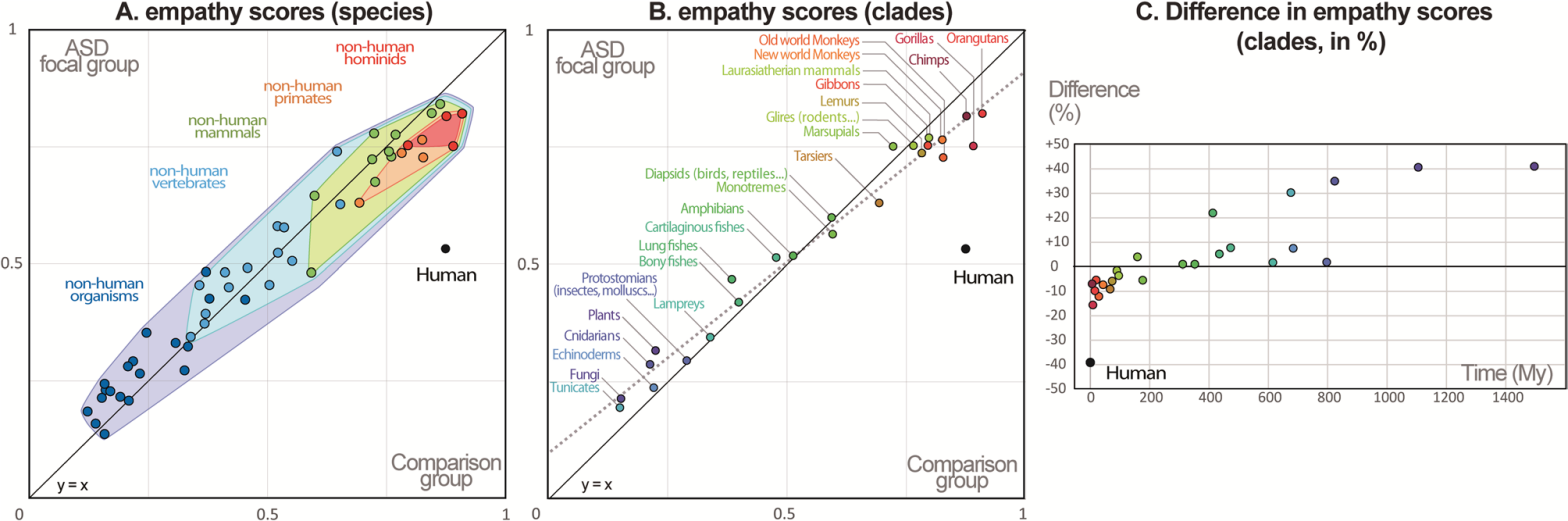
Relationships between ASD and comparison groups empathy scores: (**A**) Scores per species (n = 52). The outlines of the different polygons represent five main clades nested within each other, whereas their colored surfaces (excluding the clades they contain) are representing different non-overlapping grades (i.e., subsets of species defined according to the degree of phylogenetic divergence from human), (**B**) Scores per clade (mean of the species scores for each the 24 sister clades of the lineage leading to humans) with the corresponding regression line represented with a grey dashed line (slope excluding human scores = 0.81). While the scores attributed to the different non-human taxa are globally correlated and comparable (R^2^ = 0.976 for clade scores, excluding human species), the empathy score specifically attributed to the human species by the raters with ASD is markedly lower than that attributed by the raters of the comparison group. (**C**) Difference between scores attributed by both groups to each clade (mean of the species scores) as a function of divergence time (Mya) between them and humans.

The most notable differences between both groups concern their empathic reaction when confronted with a pair of photographs involving the human species: in the ASD group, the empathy score attributed to *Homo sapiens* (0.53) is remarkably lower than in the comparison group (0.87) (Fig. 1, Supplementary Methods S1, significance: *P* < 10^–10^). In a pair of photos containing a human, the probability to choose the human was strikingly contrasted between the two groups: overall, it decreased with divergence time for ASD raters, and increased for the comparison group (Supplementary Table S6). When the other species was a mammal, humans when chosen less than 30% of the cases for ASD raters, and more than 75% for the comparison group. For both groups, the percentage of photographic choice in favor of humans tended however to increase with the phylogenetic distances. For ASD raters, the human choice exceeded 50% only when the other species in the pair was not mammalian (Figs. 2 and 3, Supplementary Table S7).

The same contrasted result was apparent when non-human primates, or non-primate mammals, occurred in a pair: for the species most related to humans, the probability decreased for ASD raters, and increased for the comparison group (cf. Supplementary Table S6). Similarly, the slope difference between the two groups was highest when the pair involved one human (Δ slope = 2.5), then the difference narrowed as the divergence time increase relative to us (Δ slope = 1.3 for non-human primates, 1.1 for non-primate mammals, 0.4 for non-mammal vertebrates, and 0.6 for non-vertebrate organisms) (cf. Supplementary Table S8).

Finally, the change in response time according to the type of taxa presented differed between each group. Response time displayed by ASD raters was higher than in the comparison group when the pairs of photographs involved a human (an additional 1.1 s, SE = 0.724), although this trend was not significant (t = 1.458, df = 51, *P* = 0.14). This same tendency, although weakly expressed, is apparent when non-human primates, or non-primate mammals, occurred in a pair (Supplementary Figure S4).

## Discussion

### The singular indecipherability of human emotions by people with ASD… A paradox?

Our results highlight that both groups of raters present overall similar trends in their empathic preferences toward other living beings: like in the comparison group, empathy scores in the ASD focal group (i.e., probability of a species to be chosen) significantly decreased with the phylogenetic distance relatively to humans. The most striking differences between both groups concern their respective empathic reactions when confronted with a pair of photographs including humans. In the ASD group, the empathy score attributed to *Homo sapiens* (0.53, ranked 22nd out of 52 species, lowest value among viviparous mammals) is remarkably lower than in the comparison group (0.87, ranked 4^th^, close behind Orangutan, Gorilla and Chimp). Similarly, the answers of ASD raters seem to be less spontaneous than for the comparison group when the pairs of photographs involved a human being (higher average response time, although non-significant).

The empathy score specifically attributed by the ASD group to our own species is remarkably low and represents a striking outlier in the yet very sharp correlation observed between empathy score and divergence time. This result is consistent with previous studies, which emphasized that understanding human beings would be more difficult for people with ASD than decoding “animals” (mostly mammal pets)^20,22,24^. Nevertheless, the fact that for all other non-human organisms, the pattern of empathic preference of the ASD group is globally similar to that of the comparison group, is all the more surprising. It indeed shows that people with ASD have no major difficulty to perceive emotional clues that are yet, in all likelyhood, only decipherable by using a fundamentally anthropomorphic interpretative grid^7^. At the first sight, such a result may seem disconcerting, as humans are unquestionably the most anthropomorph of all living beings (tautologically, all the anthropomorphic traits present in other organisms are necessarily present in humans too). How then to explain that for the ASD group, human species didn’t score at least equally to other hominid primates, but considerably less (i.e., 0.53 versus 0.86 for other hominids), even less than most of the non-hominid mammals (0.74) or birds (0.65), scoring our species as low as cold-blooded vertebrates such as amphibians and non-avian reptiles (0.51 and 0.57, respectively)? What makes the empathic perceptions of people with ASD towards their fellow so weak and so frankly disconnected from phylogenetic proximity (which is null distance in the present case)? According to the anthropomorphic stimuli hypothesis, answering this question come down to find which singular property of human beings (whatever its nature) would be able to mute, in people with ASD, the empathizing stimuli of our species.

The Theory of mind (ToM) is an umbrella concept which refers to a person’s ability to understand another person’s mental states, such as beliefs or intentions. This concept is used to designate more precisely our abilities to take another’s perspective from a cognitive point of view (ability to infer what another is thinking), and thus differs in the literature from the notion of empathy, which is focusing on our ability to infer the emotions another is feeling. The frontier between both notions nevertheless suffers from a lack of clarity, as depending on task demands, an efficient ToM system could require emotional or cognitive perspective-taking, or both^35–37^. While the emotional register of humans may seem to most of us to be more accurate (i.e., more diversified according to the situation) and more intuitively accessible to us than that of other species, one may object that it can also be more ambivalent, as its interpretation depends on the context. Correctly reading another’s emotional behaviour (recognizing or being emotionally affected by a laughing, a crying, a frowning…) is quite different from correctly interpreting the mental state hereby expressed. Taken out of their context, and more particularly if interpreted by people with attenuated perspective-taking skills, these signals can be disconcerting or misleading (e.g., tears of joy, nervous laughs). Moreover, in different situations, whether it is to preserve his privacy, to comply with social convention, by bluffing strategy or comedy, humans are used to feign, divert, or contain their emotional expression^38,39^. In contrast, while the other species might appear to us less expressive than humans and their emotional state more difficult to interpret intuitively, their emotional expression is also likely more determinist, spontaneous and stereotyped (i.e., traits that are all the more obvious as the cognitive capacity of these organisms are lower). The mental state of an animal can thus be considered by some as relatively transparent, provided one has learnt to be attentive to weak behavioral signals and to interpret their meaning. Moreover, while a discussion with someone can make us realize that we have deeply misinterpreted his/her emotions, such a corrective feedback is less obvious with an animal. Unless a strong reaction on its part (e.g., aggressiveness, escape), its silence may support the belief we have in our ability to correctly understand it.

Social integration can be a major challenge for people with ASD, and during their lives, they may have experienced more than others the difficulties of interpretating relative’s emotional and mental states. They may have thus better internalized (consciously or not) the fact that such inferences on others human beings—unlike pets, for instance—carry a substantial part of uncertainty, and that misinterpretations can lead to unfortunate consequences. This hypothesis is not only supported by the remarkably low scores attributed to human beings by people with ASD, but also by the fact that their responses were less spontaneous (i.e., higher response times) than those in the comparison group when pairs of photographs included a human being. Such differentiation of treatment between humans and other organisms by people with ASD is consistent with the theoretical distinction formulated by Prothmann et al. in 2009 to account for the contrasting abilities of people with ASD to interact with animals, and according to which animals could be assimilated to *agents of actions* (i.e., communicating their intentions non-verbally via body language) and humans to *agent of attitudes* (i.e., using meta-representations)^28^. These results are also consistent with recent studies suggesting that ToM ability is not at a global deficit in those on the autistic spectrum but may relate to the mindreading of specifically human agents^25,40^.

Moreover, eye avoidance (i.e., reluctance or difficulty to make prolonged eye contact with another) has been shown to be associated with ASD, and has been hypothesized as a plausible explanation for their face recognition deficits^41,42^. In contrast, unlike for humans, gathering information from a mammal face (notably gazing at the eyes) and cognitively processing information from them have been shown to be relatively preserved skills in individuals with ASD^21–25^. Atherton & Cross hypothesized that people with ASD would not find human stimuli more difficult to decode but would rather find them less interesting^25^. Other studies and personal testimonies also reported that looking into another person’s eyes can be an unpleasant, even painful and perceived as socially threatening experience for a person with ASD^41–43^. Altogether, these different lines of evidence prompt us to wonder if the relative interactional preferences of people with ASD for animals would not reflect more a specific anthropophobic reaction than a generalist biophilic attitude^44,45^.

As a last word, it should be emphasized that while the difficulties of deciphering human emotions (relatively to other organisms) are exacerbated in people with ASD, they might also affect the general population in a much lesser extent. Intriguingly, while the control group (supposedly representing neurotypical individuals) gave humans one of the highest scores (0.87), it was still significantly lower than the maximum score obtained by the Orangutan (0.91), and not different from the other great ape species (Gorilla (0.89) and Chimp (0.86)). We cannot rule out the presence of a few percent of participants with ASD in the control group that would have contributed to lower the human score slightly. For example, just removing 12 judges (or 2.8%) preferring a non-human species is sufficient to get orangutan and human scores not significantly different (*P* > 0.05). However, this remains a partial explanation, because in order to get a significantly higher score for humans relatively to chimpanzees requires removing at least 49 judges (or 11.4%) preferring non-human species. Another non-exclusive explanation could lie on the peculiarities of the human pictures presented (two men and two females, each ethnically distinct), which cannot be evaluated in the context of this study. Further work is needed to understand why, in the control group, the human species is not displaying the highest score among the great apes.

### A bit too human?

In both the ASD and comparison groups, the empathy score attributed to another species overall increased with the phylogenetic proximity to us. And yet, interestingly, when compared with the comparison group, this increase in scores attributed by ASD people appears to be the less strong as species are close to us.

This phenomenon is compatible with the anthropomorphic stimuli hypothesis according to which the more anthropomorphic a species is, the more it can be cognitively processed in the same way as human. While it implies in the comparison group a regular increase of the empathy with phylogenetic proximity to humans, it might translate in the ASD group, into a relatively similar trends, although modulated for our closest relatives: The more a species is resembling to us, the more it would receive a cognitive treatment approaching the one specifically dedicated to humans, which in people with ASD would translate into a lower ability to understand its emotions.

### Human-mammal empathic comparisons as a discriminant item

Self-report instruments used to assess empathic performances in ASD are almost exclusively involving interhuman relational situations (e.g., Bryant's Empathy Index, Empathy Quotient, Interpersonal Reactivity Index, Rogers et al. 2007)^17–19,46^. Among the few exceptions, one item of Bryant's Empathy Index (on 22 in total) involves our perception of pets’ feelings, and two items of the Empathy Quotient (on 60) explores our preferences for animals over humans and our emotional reaction toward an animal in pain, respectively. Our results show however that empathic preferences of people with and without ASD differ strikingly when photographic pairs involve a comparison between a human and another mammal: a large majority of raters without ASD feel that they understand humans (feeling and emotions) better than primates (69.4%) or mammals (76.2%, including all non-human primates), while at the opposite, most of the raters with ASD consider understanding primates (78.3%) or mammals (71.7%) better. Such differences are strongly supported statistically, but further studies involving a larger sampling of comparisons will be needed to quantify their discriminatory properties and to define the species in which they are highest. Moreover, it is also important to note that participants with ASD self-reported their diagnosis. Although our analyses filtered only those who declared an ASD diagnosis made by a professional, it was not possible to verify these statements as our online protocol was based on anonymity. If this discriminatory trend would be confirmed, approaches comparing empathic preferences between humans and an optimized selection of other organisms might constitute a relevant enrichment of the existing diagnostic procedures.

## Conclusion

For more than two centuries now, we know thanks to evolutionary biology that all living species are related to each other and that humans are only an animal species among others. It is now clear that the phylogenetic proximity of an organism relatively to us is intuitively—although indirectly—accessible through our empathic perceptions and that it significantly modulates our response toward it^7^. The category of "*animals*" (used indiscriminately and in opposition to humans) thus appears not to be more relevant from a cognitivist perspective than it was for biology. For example, most of us are able to spontaneously perceive that a chimp *shares more things in common* with us than with a frog, or likewise, a frog with us more than with a snail. Earth biodiversity puts at the disposal of scientists a plethoric gradient of organisms that are more or less evolutionary close to us, more or less morphologically and behaviorally resembling us, and therefore anthropocentrically perceived and treated as being *more or less human*. For these reasons, in most of the situations, studies dealing with the human mind and its relation to the natural world (e.g., cognitive sciences, psychology, human evolutionary biology, anthropology) would benefit from abandoning the *animal* operational concept, and substituting this representation by an interpretive grid objectively accounting for evolutionary divergence with human. Such a paradigm shift could not only sharpen investigations on human interactions with the living world, but could also, as evidenced by the present work, open new opportunities to better understand the perception our own species has of itself.

## Methods

### Ethics statement

All research was performed in accordance with relevant guidelines/regulations. In accordance with the French legislation, the protocols for this study have been submitted and approved by the French National Commission on Informatics and Liberty (CNIL number 2-19061). The CNIL, an independent public authority affiliated to the *European Data Protection Board*, is responsible for ensuring that information technology ethically serves the citizen and that it does not infringe on human identity, human rights, privacy or individual or public liberties. The present research is an online experiment with volunteers interacting remotely and anonymously, thus no ethical approval was required—nor possible—from an academic or scientific comity under French legislation, as their recourse is only possible if it is compulsory. The European GDPR (General Data Protection Regulation) was fully applied, and all participants were informed of the subject of the study (perception of biodiversity by people with ASD), of the protocol for processing personal data, and of their rights of withdrawal (made possible by an individual and anonymous confidential code). For all participant, access to experiments was conditional on an explicit informed consent to participate, obtained by clicking on the button: "I understand and accept the conditions and I participate". Data were all collected and analyzed anonymously.

### Photographic stimuli

Pictures of a diversified set of 52 macroscopic eukaryote species have been selected (47 animal species—including *Homo sapiens*, four plants and one fungus, representing a total of 25 sister clades of the lineage leading to human). For reproducibility, this picture dataset was the same as in Miralles et al. 2019^7^ (cf. details in Supplementary Methods S1).

### Procedure

An online application was generated to present random pairs of photographs. The procedure used was identical to the one used in Miralles et al. (2019) to assess empathic preferences^7^. No information on the photographed individuals was given to the participants, who could therefore only base their choice on images. For each pair, the rater was instructed to click on the photograph corresponding to the answer to the following question: “*I feel like I'm better able to understand the feelings or the emotions of* [choice among a pair of pictures representing different organisms]”. The position of the photograph on the screen (left or right) was randomly ascribed for each pair and for each rater. Each rater had 22 distinct pairs of photographs to assess, randomly drawn from the set of 52 species, with the constraint that for each pair, the two species were drawn from distinct clades, and that no species is seen more than once. Three pairs, randomly chosen from among those previously viewed (excluding the last four pairs already seen), were presented again at the end to estimate judgment reliability.

### Raters sampling and diagnosis of autistic spectrum disorders

The online questionnaire (in French) was shared on the internet with the help of 26 regional autism resource centers (CRA, French public medical and social structures with a regional vocation, places of resources, information and orientation on autism, open to all) and of a dozen of associations, websites and administrators of social network groups involved in the awareness and support of autism spectrum disorders. Although it was explicitly and specifically addressed to adult people with ASD, the questionnaire was accessible to everybody. A total of 389 raters has participated to the study between June and November 2020. For each rater, the following general information was collected: *sex*, *year and month of birth*, and *nationality*. Raters were also asked whether they presented an *Autism Spectrum Disorder* (1. Yes, diagnosis made by a professional / 2. Yes, self-diagnostic / 3. No, but a diagnostic is underway / 4. No, and no diagnostic process has been carried out. / 5. No, and this has been confirmed to me by a professional). For those having positively answered to the previous question, details were requested concerning the *diagnosis* they received (1. Autism spectrum disorder (ASD) / 2. Pervasive developmental disorders (PDD) / 3. Typical autism / 4. Non-typical autism / 5. Asperger's Syndrome / 6. Other diagnosis). To respect the ethics of the research (e.g., anonymous participation), no medical proof of ASD diagnosis had been asked. In addition, each rater provided information on her/his *type of diet* (1. Omnivorous / 2. Vegetarian (fish allowed) / 3. Strictly vegetarian / 4. Vegan), and *opinion on the value of animal life* (1. None / 2. Low / 3. some but lower than human’s / 4. equal to human’s / 5. higher than human’s). See details in Supplementary Methods S2.

The following conservative selection on raters was applied. First, only raters declaring having received a diagnosis duly made by a professional have been considered (date of diagnosis and diagnostic system used not known). Raters have been pooled together for statistical analysis, whether they have been diagnosed with ASD, PDD, typical and non-typical autism or Asperger's Syndrome, because since 2015, all these categories fall indiscriminately under the Autism Spectrum Disorders according to the *Diagnostic and Statistical Manual of Mental Disorders* (DSM-5)^11^. Second, to reduce cultural heterogeneity, only European raters from a French speaking country were considered. Third, unreliable raters (i.e., with more than one incorrect answer during the test of judgment reliability), non-adult raters (lower than 18 years old), or raters with incomplete data were removed. Finally, evaluation of pair of photographs taking less than 0.2 s or more than 200 s were discarded.

A total of 202 raters were retained in the final sample of the *ASD focal group*, corresponding to 138 females (68%) and 64 males (32%). Their mean age was 37.99 ± 0.76 years old (range 18.12–69.46), with mean ages of 38.05 and 37.86 years old for females and males, respectively. Each photograph (four per species) was seen, on average, by 42.7 raters (range 32–54). Among the 202 selected raters, *Asperger's Syndrome* is the most frequently declared diagnosis (n = 137, 67.8%), followed by *ASD, with no more precision* (n = 57, 28.2%), *other diagnosis* (n = 3, 1.5%), *PDD* (n = 3, 1.5%), *typical* (n = 1, < 1%) and *non-typical autism* (n = 1, < 1%).

In order to characterize the results obtained from raters with ASD, we compared them with those obtained in a previous study open to everyone (*Comparison group,* 1134 participations, same species, pictures, online procedure and empathy driven question)^7^. The comparison group involved 707 females (62,2%) and 429 males (37,8%). Their mean age was 37.6 ± 0.39 years old (range 18.1–81.2), with mean ages 37.1 and 38.6 years old for females and males, respectively. Only the personal questionnaire differed slightly, as no specific question was intended to distinguish cognitive traits in this previous study initially open to everyone. The presence of few raters with ASD in the comparison outgroup cannot therefore be discarded. Despite this methodological limitation, we assume they represented a statistically negligible minority, as the prevalence of ASD is estimated to range from approximately 0.5 to 2% in western countries^47^. We consequently assimilated the outgroup to a population overwhelmingly (i.e., 98 to 99.5%) without ASD, and to referred to it under the term “neurotypical”.

### Statistics

To examine the influence of the phylogenetic divergence time relatively to humans, logistic regressions were used to analyze raters' decisions. The binary response variable corresponded to being chosen or not for the focal species (arbitrarily the species presented at the left position) during the presentation of each pair. Species and raters were considered random samples from a larger population of interest and were thus random-effect variables. Therefore, generalized linear mixed models with a binomial error structure were used. For each choice made by a rater, the difference between the phylogenetic divergence time with humans of the focal and the non-focal species was calculated, as provided by timetree.org^48^ (*cf.* Supplementary Methods S1).

The value of this difference was integrated into the model as the main variable of interest (*Test*). A taxonomic qualitative variable (*taxa*) was introduced as interaction terms with the variable of interest. The modalities of this variable were assigned with the following priority order: *huma* (for pair of pictures with one human), *prima* (with at least one non-human primate), *mamm* (with at least one non-primate mammal), *vert* (with at least one non-mammalian vertebrate), and *nvert* (with only non-vertebrate species). See details in Supplementary Methods S3.

To control for potential confounding effects, variables concerning the raters’ characteristics were also included in the model (after pooling some categories poorly represented) as interaction terms with the variable of interest. These confounding variables were the rater’s sex (qualitative: male, female), age (quantitative, centered), type of diet (qualitative: omnivorous, pesco-vegetarian, vegetarian), and opinion on the value of animal life relatively to humans (qualitative: lower, equal, higher). The significance of each independent variable was calculated by removing it from the full model and comparing the resulting variation in deviance using a Chi square test. A score was computed for each species, computed as the number of times the species was chosen, divided by the number of times it was presented to raters. A difference between two scores (e.g. for species A and B) was tested using a Fisher exact test on a 2 × 2 contingency table (A & B vs chosen & not-chosen). When A and B were from the same dataset (e.g. human and orangutan from the control group), comparisons involving both A and B were excluded. To examine the influence of the phylogenetic divergence time on rater response time, linear regressions were used. Species and raters were considered random samples from a larger population of interest and were thus random-effect variables. Therefore, mixed models with a Gaussian error structure were used. A qualitative variable describing the two groups (ASD and control) was also introduced, as well as the taxonomic qualitative variable *taxa*, and their interactions. These computations were done using the lme4 package on R 3.6.3 software^49,50^.

## Supplementary Information

Supplementary Information.

## Data Availability

Data supporting the findings of this work are available within the paper and its Supplementary Information files.
