# Supplementary material for: Self-perceived empathic abilities of people with autism towards living beings mostly differs for humans
Source: Sci Rep. 2022 Apr 15;12:6300. doi: 10.1038/s41598-022-10353-2 (PMC9012821; doi:10.1038/s41598-022-10353-2)

Supplementary Information

Article. Self-perceived empathic abilities of people with autism towards living beings mostly differs for humans.

Supplementary Methods S1 : Photographic sampling and specific empathy scores. Pictures of a diversified set of 52 macroscopic eukaryote species have been selected, representing 47 animal species - including *Homo sapiens*, four plants and one fungi (same photographic sampling as in Miralles et al. 2019, cf. sampling details and empathy scores in the Table S1 and Figure S1 thereafter). Although any species sampling involved in a comparative study of the diversity of life on a large scale inevitably has an arbitrary component, our sampling has been developed in order:

(1)To optimize the representativeness in terms of phylogenetic diversity, which translates here into the representativeness in terms of temporal divergence from humans, given the hypothesis to be tested: In that respect, most of the clades connecting at different level of the tree of life and that are placed as sister clades of the lineage leading to humans are represented (Hedges et al. 2015, Kumar et al. 2017). Nevertheless, microscopic organisms have been excluded despite the fact they make up a considerable part of the biodiversity, because we considered them to be beyond our common sensory reach. In total, and excluding *H. sapiens*, our sampling represent 24 clades that diverged from the lineage leading to man at different times, from our sister clade (chimpanzee, 6.7 Mya) to the very distantly related plants clade (1496 Mya) (Kumar et al. 2017).

(2) To optimize the representativeness of the phenotypic and phylogenetic diversity among each of these clades: Most of them are represented by several species that have been selected to be highly divergent from each other (i.e. intra-clade divergence time values are always ≥45 million years, Kumar et al. 2017). Species selected for a given clade can therefore be considered as different taxonomic samples (i.e. replicates) in order to measure the variability of our empathic reactions for a given divergence time value. Eleven poorly diversified clades (most often very closely related to humans) are represented by a single species (ex. Panina, Gorillini, Ponginae) whereas up to eight highly divergent species have been selected in order to take into account polymorphism of hyperdiversified lineages such as protostomians. For this particular clade, we have for instance selected three very divergent mollusks (a snail, a cuttlefish and a scallop), one annelid (an earthworm) and four very different arthropods (a beetle, a shrimp, a spider and a tick). Given that domestic species have been transformed by human selection, they have been excluded from the sampling because it is likely that their evolution have been directionally driven by our empathic or aesthetical preferences. As far as possible, species overrepresented in the media and entertainment (e. g. bottlenose dolphin) have been avoided or replaced by closely related species that are less popular (e.g. beluga whale).

(3) To take into account the variability among each species: For each species, four distinct photographs of distinct living individuals have been selected from online open sources in

order to represent phenotypic variation of living individuals and minimize the enhancement bias specific to each shot (N total = 208 photos). Only photographs representing adult individuals were selected, as it has been shown that in mammals juvenile traits can positively influence our empathic perceptions (Prugda & Neumann 2014, Borgi & Cirulli 2015). For humans, two women and two men representing four distinct ethnic phenotypes have been selected.

Table S1: Photographic sampling and specific empathy scores.

**Clade (N species sampled)**	**Divergence time (My)**	**Species**	**Common name**	**Empathy scores**	
				**ASD focal group** (n=202 raters, present study)	**Comparison group** (n=1134 raters, from Miralles et al. 2019)
**Hominina (1)**	0	*Homo sapiens*	Human	0.532	0.874
**Panina (1)**	6,65	*Pan troglodytes*	Chimpanzee	0.815	0.876
**Gorillini (1)**	9,06	*Gorilla gorilla*	Gorilla	0.751	0.890
**Ponginae (1)**	15,76	*Pongo sp.*	Orangutan	0.821	0.909
**Hylobatidae (1)**	20,19	*Hylobates lar*	Lar Gibbon	0.753	0.795
**Cercopithecidae (1)**	29,4	*Macaca fuscata*	Japanese macaque	0.727	0.827
**Platyrrhini (1)**	43,2	*Cebus capucinus*	Capuchin	0.765	0.825
**Tarsiiformes (1)**	67,1	*Tarsius sp.*	Tarsier	0.630	0.693
**Strepsirrhini (1)**	74	*Lemur catta*	Ring-tailed lemur	0.737	0.782
**Glires (2)**	90	*Lepus sp.*	Hare	0.729	0.760
—	—	*Sciurus vulgaris*	Red Squirrel	0.776	0.769
**Laurasiatheria (4)**	96	*Antidorcas marsupialis*	Springbok antelope	0.675	0.726
—	—	*Ursus arctos*	Brown bear	0.822	0.845
—	—	*Delphinapterus leucas*	Beluga whale	0.740	0.755
—	—	*Vulpes vulpes*	Red Fox	0.841	0.863
**Marsupialia (2)**	159	*Phascolarctos cinereus*	Koala	0.778	0.724
—	—	*Macropus rufogriseus*	Benett’s wallaby	0.723	0.720
**Monotremata (2)**	177	*Tachyglossus sp.*	Echidna	0.645	0.599
—	—	*Ornithorhynchus anatinus*	Platypus	0.482	0.592
**Diapsida (4)**	312	*Alligator mississippiensis*	Mississipi alligator	0.524	0.522
—	—	*Turdus merula*	Common blackbird	0.627	0.653
—	—	*Teira duguesi*	Madeiran wall Lizard	0.506	0.552
—	—	*Testudo hermanni*	Hermann’s tortoise	0.740	0.646
**Amphibia (2)**	352	*Rhinella marina*	Cane toad	0.455	0.504
—	—	*Hyla arborea*	European tree frog	0.580	0.521
**Dipnoi (2)**	413	*Neoceratodus forsteri*	Queensland Lungfish	0.455	0.357
—	—	*Protopterus amphibius*	Gilled Lungfish	0.482	0.411
**Actinopterygii (3)**	435	*Sphyraena sp.*	Barracuda	0.373	0.368
—	—	*Amphiprion ocellaris*	Common clownfish	0.491	0.458
—	—	*Danio rerio*	Zebrafish	0.394	0.370
**Chondrichthyes (2)**	473	*Scyliorhinus canicula*	Small-spotted catshark	0.450	0.419
—	—	*Carcharodon carcharias*	Great white shark	0.577	0.535
**Agnatha (1)**	615	*Petromyzon marinus*	Sea lamprey	0.345	0.339
**Tunicata (2)**	676	*Clavelina caerulea*	Blue sea squirt	0.231	0.160
—	—	*Ciona edwardsi*	Yellow sea squirt	0.159	0.139
**Echinodermata (2)**	684	*Pisaster ochraceus*	Ochre starfish	0.266	0.232
—	—	*Paracentrotus lividus*	Purple sea Urchin	0.208	0.209
**Protostomia (8)**	797	*Timarcha sp.*	Bloody-nosed beetle	0.273	0.326
—	—	*Sepia officinalis*	Common cuttlefish	0.427	0.453
—	—	*Lumbricus sp.*	Earthworm	0.216	0.191
—	—	*Odontodactylus scyllarus*	Peacock mantis shrimp	0.324	0.333
—	—	*Pecten maximus*	Great scallop	0.228	0.170
—	—	*Helix pomatia*	Burgundy snail	0.426	0.378
—	—	*Araneus diadematus*	Diadem spider	0.331	0.307
—	—	Ixodida *gen. sp.*	Tick	0.137	0.158
**Cnidaria (2)**	824	*Actinostola sp.*	Sea Anemone	0.292	0.218
—	—	*Chrysaora sp.*	Jelly Fish	0.282	0.207
**Fungi (1)**	1105	*Boletus edulis*	Cep mushroom	0.214	0.152
**Plantae (4)**	1496	*Echinocactus sp.*	Barrel cactus	0.244	0.158
—	—	*Quercus sp.*	Oak tree	0.483	0.371
—	—	*Fucus vesiculosus*	Rockweed	0.185	0.122
—	—	*Rosa sp.*	Rosebush	0.353	0.246

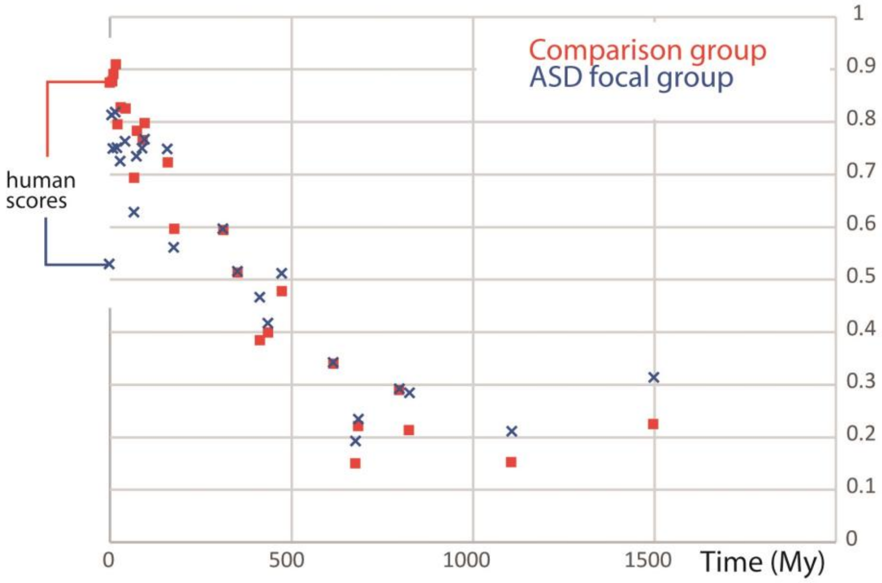

Figure S1A. Mean empathy scores attributed to each clade as a function of divergence time between them and humans (each mean score was calculated from the scores of the species belonging to that clade). See the figure 2 in the main text for a similar representation including empathy scores for each species.

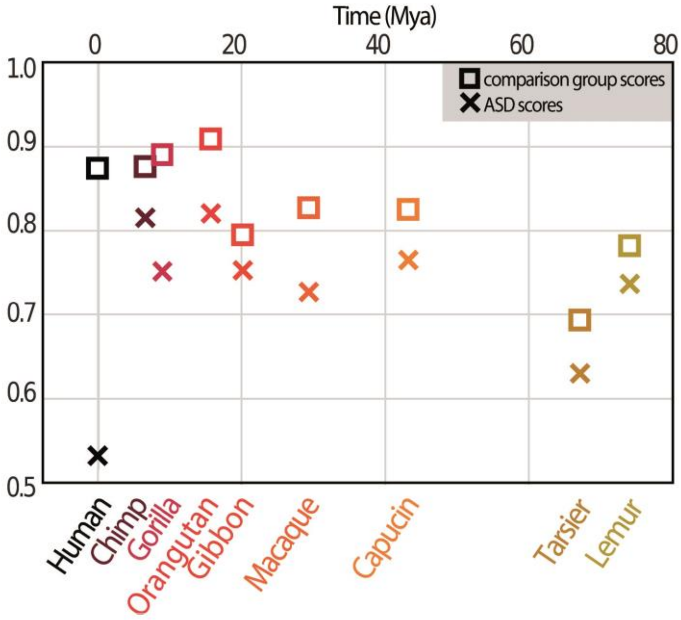

Figure S1B. Empathy scores attributed to each sampled primate species as a function of divergence time (Mya) between them and humans (detail from the Figure S1A above).

References:

Prguda. A. & Neumann. D. L. (2014). Inter-human and animal-directed empathy: A test for evolutionary biases in empathetic responding. *Behav. Process.* **108**. 80–86. https://doi.org/10.1016/j.beproc.2014.09.012.

Borgi. M. & Cirulli. F. (2015). Attitudes toward Animals among Kindergarten Children: Species Preferences. *Anthrozoös* **28**. 45–59. https://doi.org/10.2752/089279315X14129350721939.

Hedges. S. B.. Marin. J.. Suleski. M.. Paymer. M. & Kumar. S. (2015). Tree of life reveals clock-like speciation and diversification. *Mol. Biol. Evol.* **32**. 835–845. https://doi.org/10.1093/molbev/msv037.

Kumar. S.. Stecher. G.. Suleski. M. & Hedges. S. B. (2017). TimeTree: A Resource for Timelines. Timetrees. and Divergence Times. *Mol. Biol. Evol.* **34**. 1812–1819. https://doi.org/10.1093/molbev/msx116.

Miralles, A., Raymond, M. & Lecointre, G. (2019) Empathy and compassion toward other species decrease with evolutionary divergence time. *Sci Rep* **9,** 19555. https://doi.org/10.1038/s41598-019-56006-9

Supplementary Methods S2. Questionnaire (original questions in French in blue) (photos by A. Miralles)

Personal informations questionary:

What is your sex ? / *Quel est votre sexe ?*
□ Female / *Femme*
□ Male / *Homme*

What is your year and month of birth ?/ *Quels sont votre année et mois de naissance ?*

What is your nationality ? / *Quelle est votre nationalité ?*

Do you show an Austism Spectrum Disorder ? / Presentez vous un Trouble du spectre de l'autisme ?
□ Yes, self-diagnostic / Oui, autodiagnostic
□ No, but a diagnostic is underway / Non, mais un diagnostic est en cours
□ No, and no diagnostic process has been carried out / Non, et aucune démarche diagnostique n'a été menée
□ No, and this has been confirmed to me by a professional / Non, et cela m'a été confirmé par un professional

If the answer is yes, could you specify the diagnostic ? / Si vous avez répondu oui à la question précédente, pouvez-vous préciser lequel ?:
□ Autism spectrum disorder (ASD) / Trouble du spectre autistique (TSA)
□ Pervasive developmental disorders (PDD) / Troubles envahissants du développement (TED)
□ Typical autism / Autisme typique
□ Asperger's Syndrome / Syndrome d'Asperger
□ Non typical autism / Autisme atypique
□ Other diagnostic / Autre diagnostic

What is your diet ? / *Quel est votre régime alimentaire ?*
□ Omnivorous (I eat everything) / *Omnivore (je mange de tout)*
□ Pesco-vegetarian (no meat, but I can eat fish) */ Pesco-végétarien (pas de viande, mais je m'autorise le poisson)*
□ Vegetarian (no meat nor fish) / *Végétarien (ni viande ni poisson)*
□ Vegetalian/vegan (no animal products at all) / *Végétalien/Vegan (aucun produit d'origine animale)*

In my opinion, the life of an animal.... / *Selon moi, la vie d'un animal…*
□ has no value / *n'a aucune valeur.*
□ has little value / *a peu de valeur.*
□ has value, but that of a human being is superior to it / *a de la valeur, mais celle d'un être humain lui est supérieure.*
□ is equal to that of a human being / *est égale à celle d'un être humain.*
□ is superior to that of a human being / *est supérieure à celle d'un être humain.*

Photographic questionnaire:

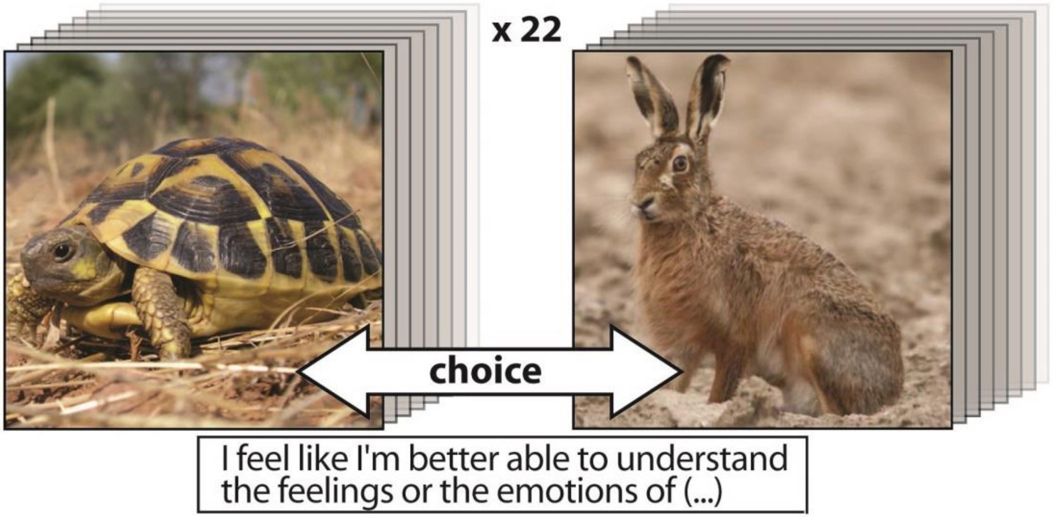

J'ai l'impression de mieux reconnaitre / comprendre les sentiments / émotions de (…)

Supplementary Methods S3. Taxonomic qualitative variable (*taxa*) introduced as interaction terms with the variable of interest. The Modalities of the qualitative taxonomic variable (*taxa*) are defined on subsets from a selection of complementary (non-redundant) photographic pairs. These subsets have been designed to represent non-overlapping paraphyletic categories in order to discriminate the respective effects of these "phylogenetic segments" (more or less divergent from human) on rater's choices. *huma* : effect of humans, *prim* : effect of other primates species (on a dataset excluding humans), *mamm* : effect of distantly related mammals (i.e. excluding primates), *vert* : effect of distantly related vertebrates (i.e. excluding mammals), *nvert* : effect of all the other organisms (excluding vertebrates). Cf. figure below.

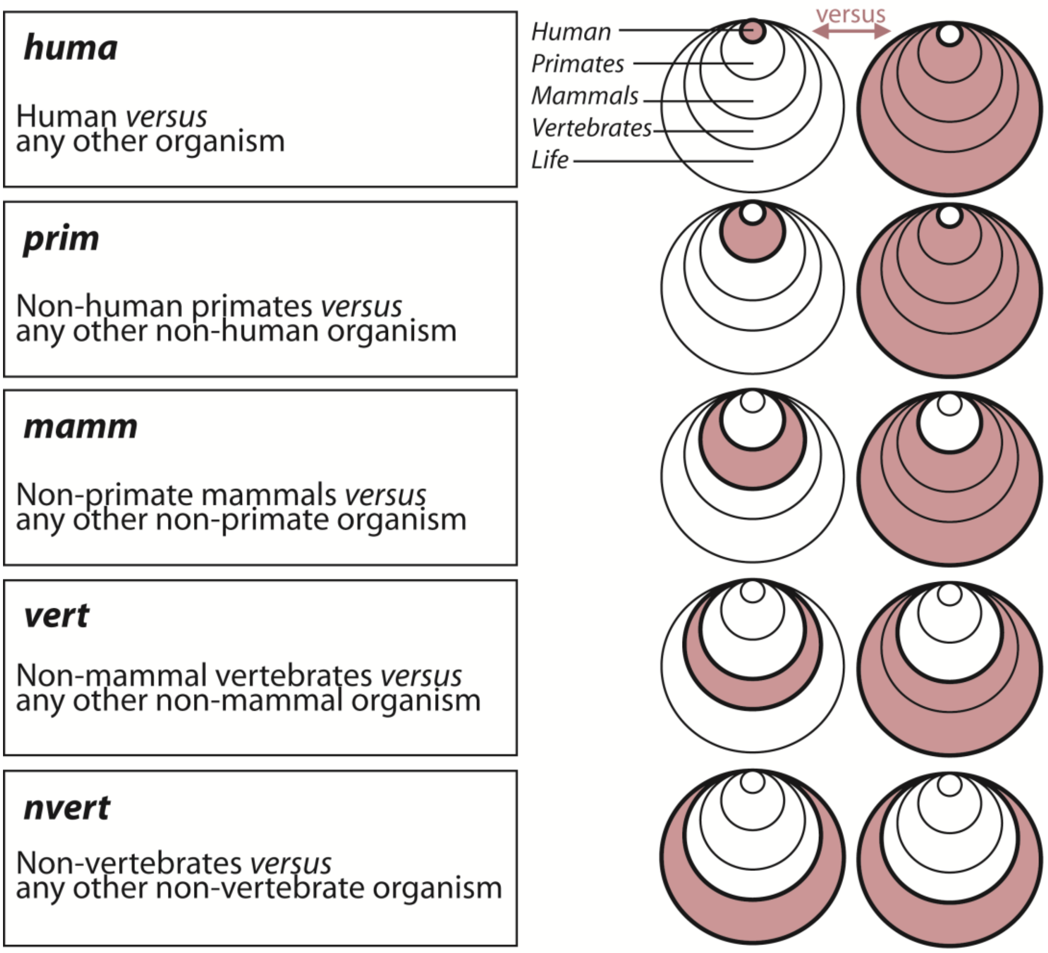

Supplementary Figure S4: Predicted participants' response time (s) as a function of the absolute divergence time (My) between the two species presented in each pair (area depicts the 95% confidence interval, n responses = 4443 for the ASD focal group and 25001 for the comparison group). *Huma*: based on pairs of photographs involving a human, *prima*: involving at least one non-human primate species (and excluding humans), *mamm*: involving at least one non-primate mammals species (and excluding primate species). See methodological details in Material and methods and Supplementary Methods S3.

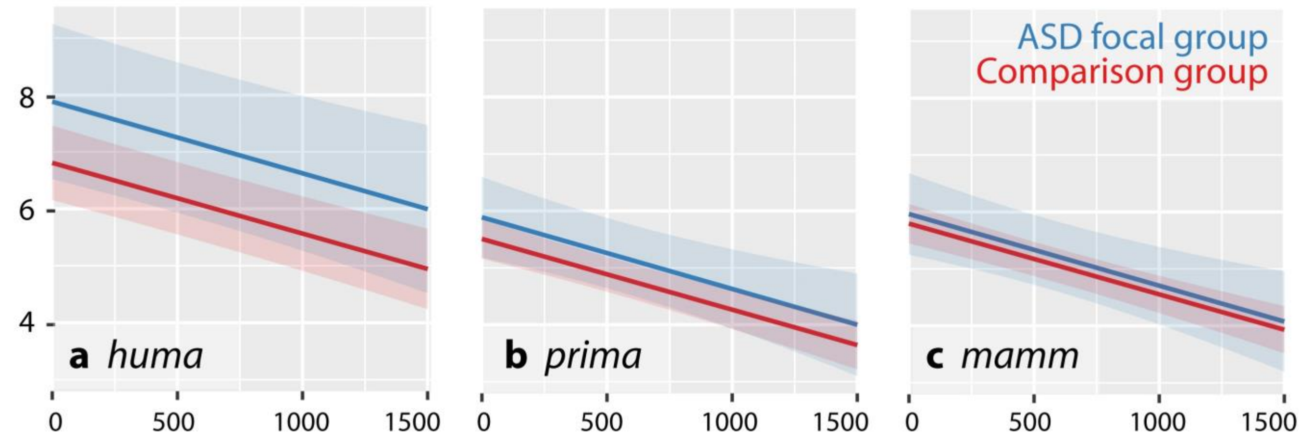

Supplementary Table S5. Analysis of deviance table (type II Wald chisquare tests). Effect of each variable on the choice made by raters from the ASD focal group (A), comparison group (B) and the whole data-set (C); Bold characters indicate significant ($P < 0.05$) values.

A	ASD focal group		
**Fixed effects**	**Chisq**	**Df**	**P-value**
Test	193.1	1	**$<2.2 \times 10^{-16}$**
Interaction with:			
Sex	0.54	1	0.462
Age	0.10	1	0.755
Diet	0.47	2	0.792
Life value	10.47	2	**0.005**

B	Comparison group		
**Fixed effects**	**Chisq**	**Df**	**P-value**
Test	921.7	1	**$<2.2 \times 10^{-16}$**
Interaction with:			
Sex	5.24	1	**0.022**
Age	11.4	1	**0.001**
Diet	1.24	2	0.536
Life value	48.7	2	**2.7×10^{-11}**

C	Total group		
**Fixed effects**	**Chisq**	**Df**	**P-value**
Test	828.80	1	**$<2.2 \times 10^{-16}$**
Interaction with:			
Sex	3.30	1	0.069
Age	11.97	1	**0.0005**
Diet	0.65	2	0.722
Life value	53.48	2	**2.4×10^{-12}**
ASD	40.15	1	**2.3×10^{-10}**
Taxa	569.28	4	**$<2.2 \times 10^{-16}$**
Taxa ASD	67.55	4	**7.5×10^{-14}**

Supplementary Table S6. Empathy model summary in (A) the ASD focal group, (B) the comparison group and (C) the whole data-set. The phylogenetic effect is described by the variable 'test', and the confounding variables (sociological and cognitive traits of participants, organisms phylogenetic divergence from human) are in interaction with 'test".

	A. **ASD focal group**				B. **Comparison group**			
**Fixed effects**	**Estimate**	**SE**	**z-value**	**Pr(>|z|)**	**Estimate**	**SE**	**z-value**	**Pr(>|z|)**
(Intercept)	0.006	0.112	0.0534	0.957	0.111	0.158	0.705	0.481
Test	1.592	0.169	9.435	**$<2x10^{-16}$**	2.536	0.187	13.606	**$<2x10^{-16}$**
Interaction with:								
Sex (male)	-0.172	0.233	-0.736	0.462	0.240	0.105	2.288	**0.022**
Age	0.003	0.010	0.312	0.755	-0.0129	0.004	-3.373	**0.001**
Diet pesco	-0.284	0.424	-0.670	0.503	-0.0109	0.192	-0.056	0.955
Diet : vege	0.010	0.301	0.034	0.973	-0.196	0.176	-1.113	0.266
Life : human >animal	0.870	0.277	3.145	**0.002**	0.610	0.113	5.406	**$6.4x10^{-8}$**
Life : animal > human	-0.025	0.322	-0.077	0.938	-0.685	0.195	-3.517	**$4.4x10^{-3}$**

	**C. Total group**			
**Fixed effects**	**Estimate**	**SE**	**z-value**	**Pr(>|z|)**
(Intercept)	-0.022	0.096	0.234	0.815
Test	0.601	0.147	4.085	**0.00004**
Interaction with:				
Sex (male)	0.207	0.114	1.817	0.069
Age	-0.014	0.004	3.459	**0.00054**
Diet pesco	-0.167	0.208	-0.802	0.422
Diet : vege	-0.030	0.177	0.169	0.866
Life : human >animal	0.743	0.125	5.962	**$2.5x10^{-9}$**
Life : animal > human	-0.630	0.201	3.130	**0.001**
ASD	-0.650	0.211	3.077	**0.002**
Taxa huma	2.592	0.233	11.137	**$<2x10^{-16}$**
Taxa prima	3.060	0.144	21.241	**$<2x10^{-16}$**
Taxa mamm	2.776	0.132	20.956	**$<2x10^{-16}$**
Taxa vert	1.491	0.118	12.566	**$<2x10^{-16}$**
Taxa huma ASD	-1.854	0.333	-5.574	**$2.5x10^{-8}$**
Taxa prima ASD	-0.632	0.216	-2.926	**0.003**
Taxa mamm ASD	-0.472	0.207	2.274	**0.023**
Taxa vert ASD	0.265	0.207	1.285	0.199

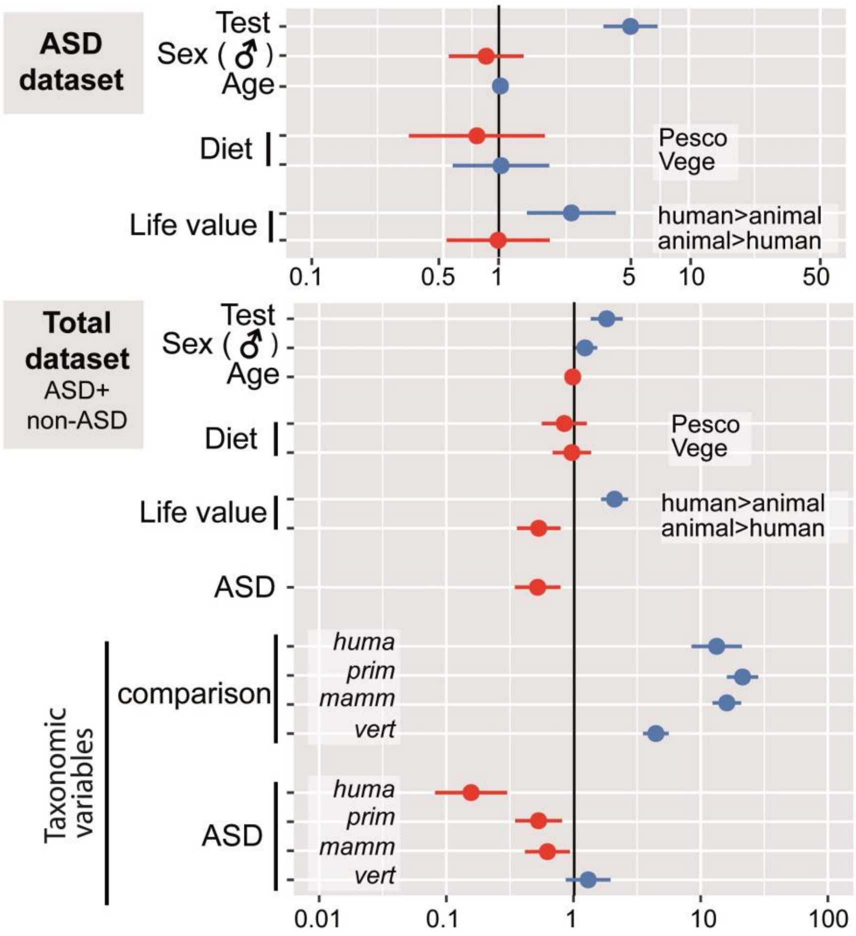

Figure S6. Effect of confounding variables. Odds ratio (for a qualitative variable: ratio of the odds of choosing the most phylogenetically related species in the depicted factor level to the odds of it occurring in the reference factor level; for age, centered variable: ratio of the odds of choosing the most phylogenetically related species in age 1 to the odds of it occurring in age 0) are represented by dots and 95% confidence interval by lines; blue or red dots indicate variables linked with an increased or decreased, respectively, choice probability for the most phylogenetically related species (n raters = 202 and 1134 in the ASD focal group and the comparison group, respectively).

Supplementary Table S7. Percentage of human chosen in pairs involving also another species. Due to the limited number of occurrences (N= 171 and 872 pairs of photographs in the ASD focal group and the comparison group, respectively), species have been hierarchically pooled into various non-overlapping paraphyletic categories reflecting different degree of phylogenetic divergence from humans. P > 0.05 (ns : non-significant), P ≤ 0.05 (*), P ≤ 0.01 (**), P ≤ 0.001 (***), P ≤ 0.0001 (****)

Human versus	TSA focal group	Comparison group	p-value (exact Fisher's exact) Odd ratio [95% confidence interval]	
**I. All mammals** (from 6 to 177 My)	28.3% (N = 17/60)	76.2% (N = 257/337)	1.61×10^{-12} 0.124 [0.063 ; 0.236]	****
**I.1. all primates** (divergence from 6 to 72 My)	21.7% (N = 5/23)	69.4% (N = 100/144)	2.756×10^{-5} 0.124 [0.034 ; 0.374]	****
**I.1.a. other hominids** (Chimp, Gorilla, Orangutan, Gibbon / divergence from 6 to 20 My)	21.4% (N = 3/14)	70.0% (N = 56/80)	1.628×10^{-3} 0.120 [0.020 ; 0.507]	**
**I.1.b. other (non-hominid) primates** (Macaque, Capucin, Tarsier, Lemur / from 29 to 72 My)	22.2% (N = 2/9)	68.7% (N = 44/64)	1.057×10^{-2} 0.134 [0.013 ; 0.785]	*
**I.2. non-primate mammals** (Glires, Laurasitheria, Marsupials, Monotrems / from 90 to 177 My)	32.4% (N = 12/37)	81.3% (N = 157/193)	9.061×10^{-9} 0.112 [0.046 ; 0.255]	****
**II. non-mammalian tetrapods** (Diapsids, Amphibians, /from 312 to 352 My)	58.8% (N = 10/17)	91.8% (N = 123/134)	9.884×10^{-4} 0.131 [0.036 ; 0.487]	***
**III. "fishes"** (Lung fishes, bony and cartilaginous fishes, Lamprey/ from 413 to 615 My)	55.0% (N = 11/20)	91.8% (N = 133/145)	1.097×10^{-4} 0.113 [0.034 ; 0.372]	***
**IV. "invertebrates"** (Tunicates, Echinoderms, arthropods, molluscs, annelids, Cnidarians / from 676 to 824 My)	70.2% (N = 40/57)	92.8% (N = 274/295)	8.220×10^{-6} 0.182 [0.083 ; 0.400]	****
**V. non-animal organisms** (fungi, plants / from 1105 to 1496 My)	75.0% (N = 12/16)	91.3% (N = 84/92)	0.076 0.290 [0.065 ; 1.520]	ns

Supplementary Table S8. Slopes of the time-empathy score curves calculated for both raters groups and for each subset of photographic comparisons. The corresponding slopes values are visualized on the figure below. *huma* : slopes for pair of pictures with one human, *prima :* for pair of pictures with at least one non-human primate and no human), *mamm* : for pair of pictures with at least one non-primate mammal and no primates, *vert* : for pair of pictures with at least one non-mammalian vertebrate and no mammals, and *nvert* : for pair of pictures with only non-vertebrate species.

subsets	*huma*	*prima*	*mamm*	*vert*	*nvert*
Comparison group (a)	3.193	3.661	3.378	2.093	0.601
ASD focal group (b)	0.688	2.379	2.256	1.709	-0.049
Difference (b - a)	-2.505	-1.282	-1.122	-0.384	-0.650

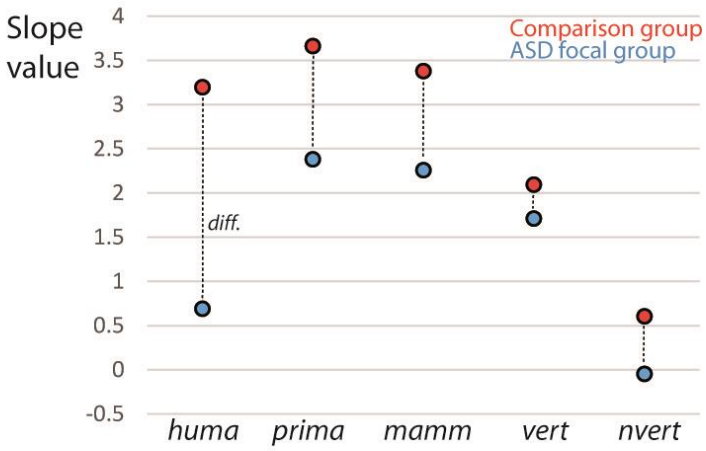

Supplement: Supplementary file 1 — Supplementary Information. [file 41598_2022_10353_MOESM1_ESM.pdf]
